# Supplementary material for: Myosin forces elicit an F-actin structural landscape that mediates mechanosensitive protein recognition
Source: bioRxiv. 2024 Aug 17:2024.08.15.608188. Preprint. [Version 1] doi: 10.1101/2024.08.15.608188 (PMC11343212; doi:10.1101/2024.08.15.608188)
Supplement: 9 [file NIHPP2024.08.15.608188v1-supplement-9.pdf]

bioRxiv preprint doi: https://doi.org/10.1101/2024.08.15.608188; this version posted August 17, 2024. The copyright holder for this preprint (which was not certified by peer review) is the author/funder, who has granted bioRxiv a license to display the preprint in perpetuity. It is made available under aCC-BY 4.0 International license.

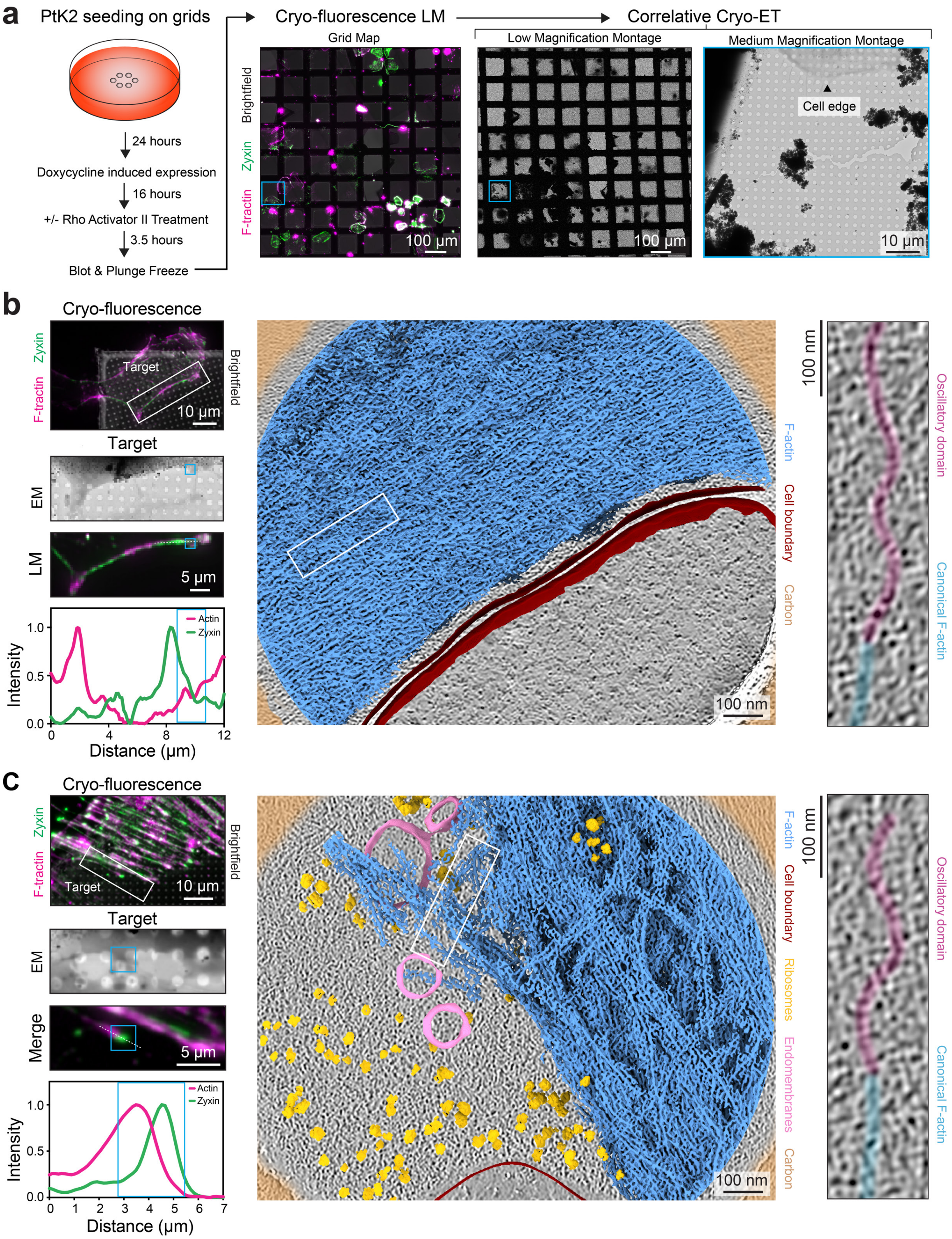

Fig. S1: Correlative cryo-fluorescence and cryo-electron tomography.
a, Schematic of sample preparation and data collection workflow. **b and c**, Additional examples of oscillatory domains imaged in zyxin-enriched adhesion sites from N = 2 independent experiments. The site in **b** features parallel bundled F-actin and was visualized in a cell not treated with Rho Activator II, while the site in **c** features more disorganized F-actin and was visualized in a cell treated with Rho Activator II. Correlative LM / EM and tomograms / segmentations are presented as in Fig. 1a,b.

bioRxiv preprint doi: https://doi.org/10.1101/2024.08.15.608188; this version posted August 17, 2024. The copyright holder for this preprint (which was not certified by peer review) is the author/funder, who has granted bioRxiv a license to display the preprint in perpetuity. It is made available under aCC-BY 4.0 International license.

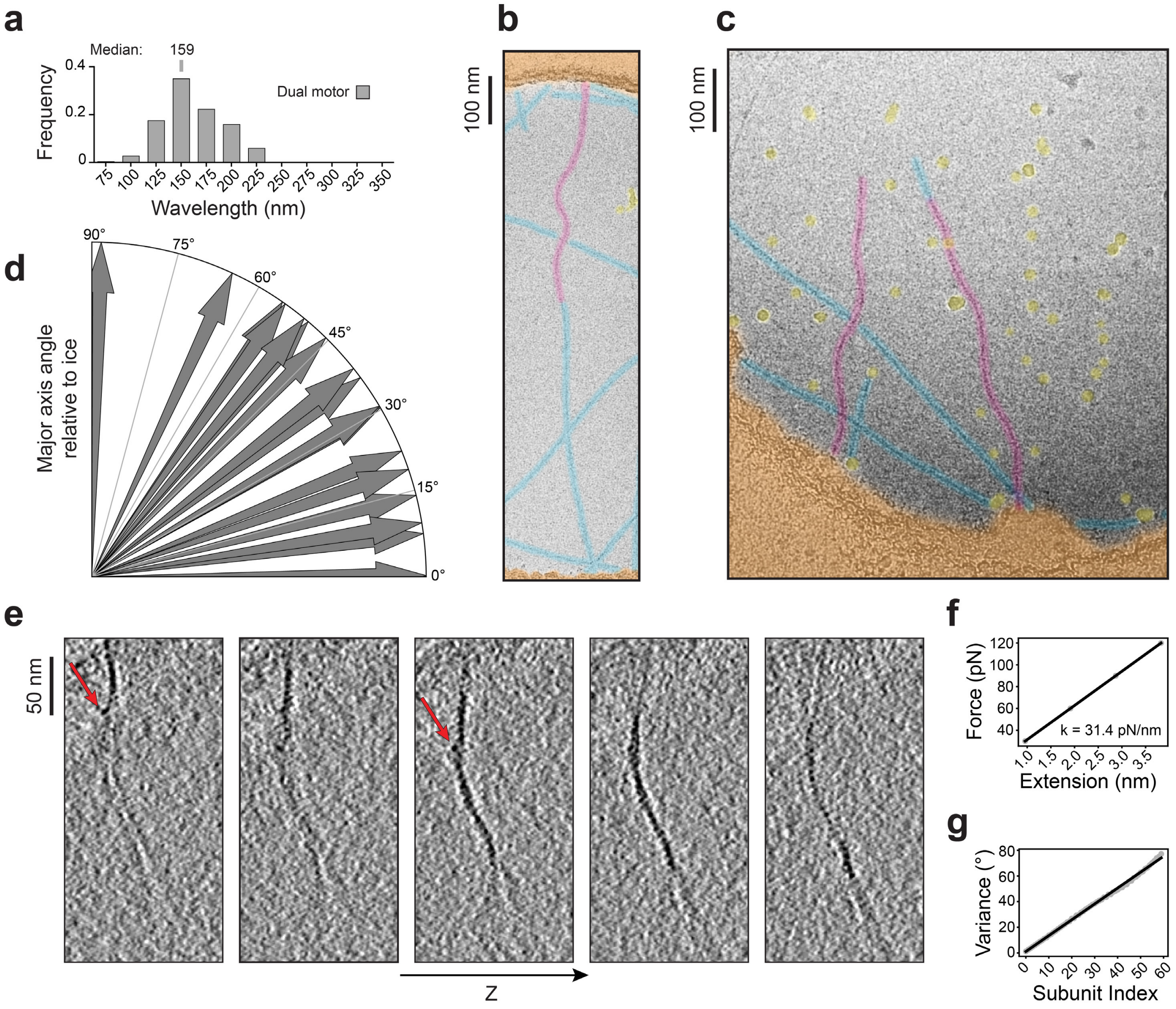

Fig. S2: Analysis of F-actin oscillatory domain morphology.
a, Quantification of oscillatory domain wavelengths in the dual motor condition. n = 251 from N = 3 independent experiments. **b and c**, False-colored cryo-EM images of the dual motor condition in the presence of ATP, displaying oscillatory domains in a hole-spanning filament (**b**) and in a pair of broken filaments (**c**). Oscillatory domains, magenta; canonical F-actin, blue; carbon film, orange; ice contamination, yellow. **d**, Polar arrow plot of oscillatory domain major axis orientation relative to the ice plane from n = 16 observations, where 0° corresponds to the major axis being parallel to the ice plane. **e**, Serial Z slices through an oscillatory domain tomogram (barbed-end directed force condition) featuring protruding densities consistent with subunit dislocations (red arrows). **f**, Calibration of the F-actin force-extension curve to determine harmonic bond stiffness in coarse-grained molecular dynamics simulations. **g**, Calibration of cumulative twist variance to determine the bending constants of the harmonic angle potential and the dihedral potential used in coarse-grained molecular dynamics simulations.

bioRxiv preprint doi: https://doi.org/10.1101/2024.08.15.608188; this version posted August 17, 2024. The copyright holder for this preprint (which was not certified by peer review) is the author/funder, who has granted bioRxiv a license to display the preprint in perpetuity. It is made available under aCC-BY 4.0 International license.

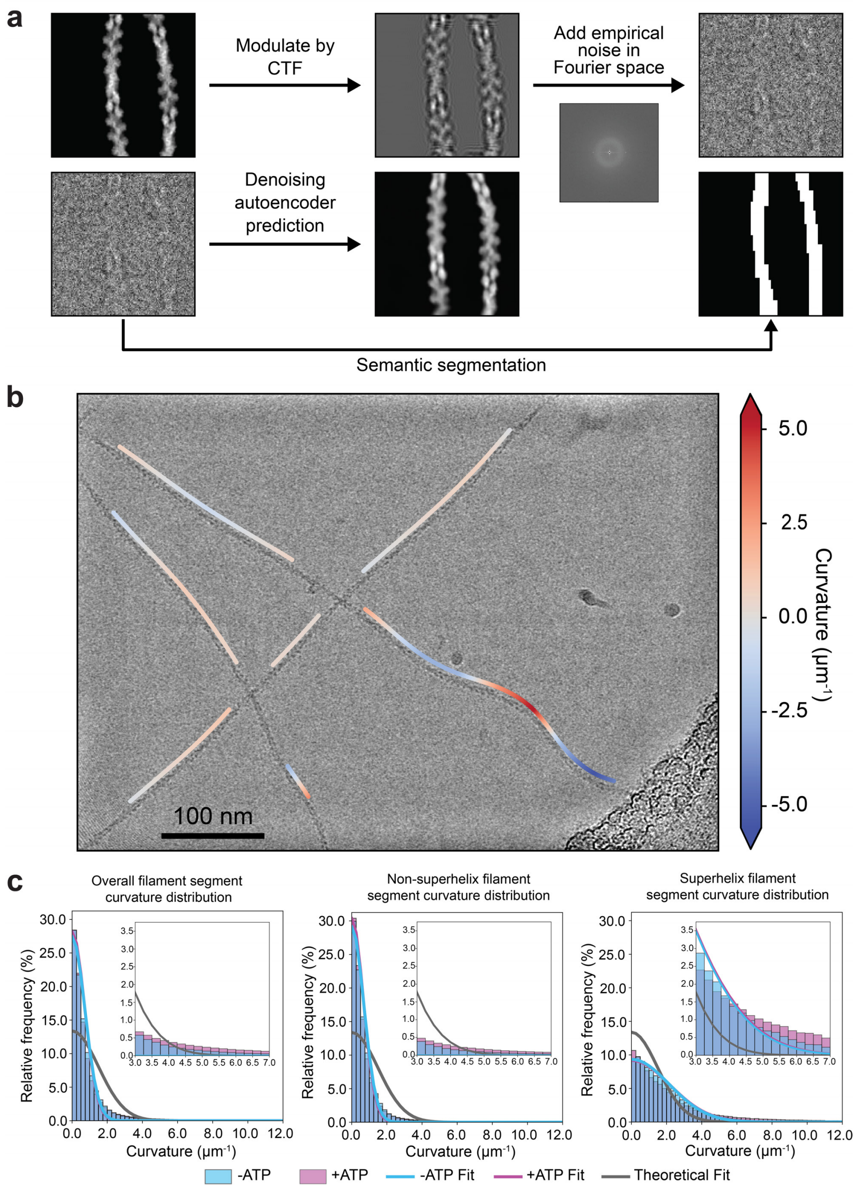

Fig. S3: Adaptation of neural network picker and filament curvature analysis.
a, Workflow for network training and subsequent picking. A synthetic particle dataset is generated by projecting PDB models of filaments featuring different computationally-generated curvatures, which are modulated by the CTF followed by the addition of a pink noise box (top). The network is then trained to denoise these noisy particles, which is then used as an input for semantic segmentation. After training with synthetic data, the network can be used to denoise and segment real data. **b**, Sample micrograph containing both straight filaments and filaments featuring oscillating curvature. Filaments are assigned estimated signed curvature values and categorized for subsequent selection. Traces are offset by one F-actin width for visualization. **c**, Curvature distributions of filaments identified in the dual myosin-motor evoked force condition (pink) or the –ATP control (blue). Measured curvature distributions of all picked filament segments (left), non-superhelical segments (middle), and superhelical segments (right) are shown as histograms, and modeled thermal bending fluctuation distributions are overlaid as blue, pink, and grey curves. The gray curve corresponds to a simple bending model described by equations 5-7 (Methods) using actin's experimentally determined persistence length of 9 μm (ref. 63), while the pink and blue curves correspond to a model fitted with a multiplicative adjustment factor as described in detail in the Methods.

bioRxiv preprint doi: https://doi.org/10.1101/2024.08.15.608188; this version posted August 17, 2024. The copyright holder for this preprint (which was not certified by peer review) is the author/funder, who has granted bioRxiv a license to display the preprint in perpetuity. It is made available under aCC-BY 4.0 International license.

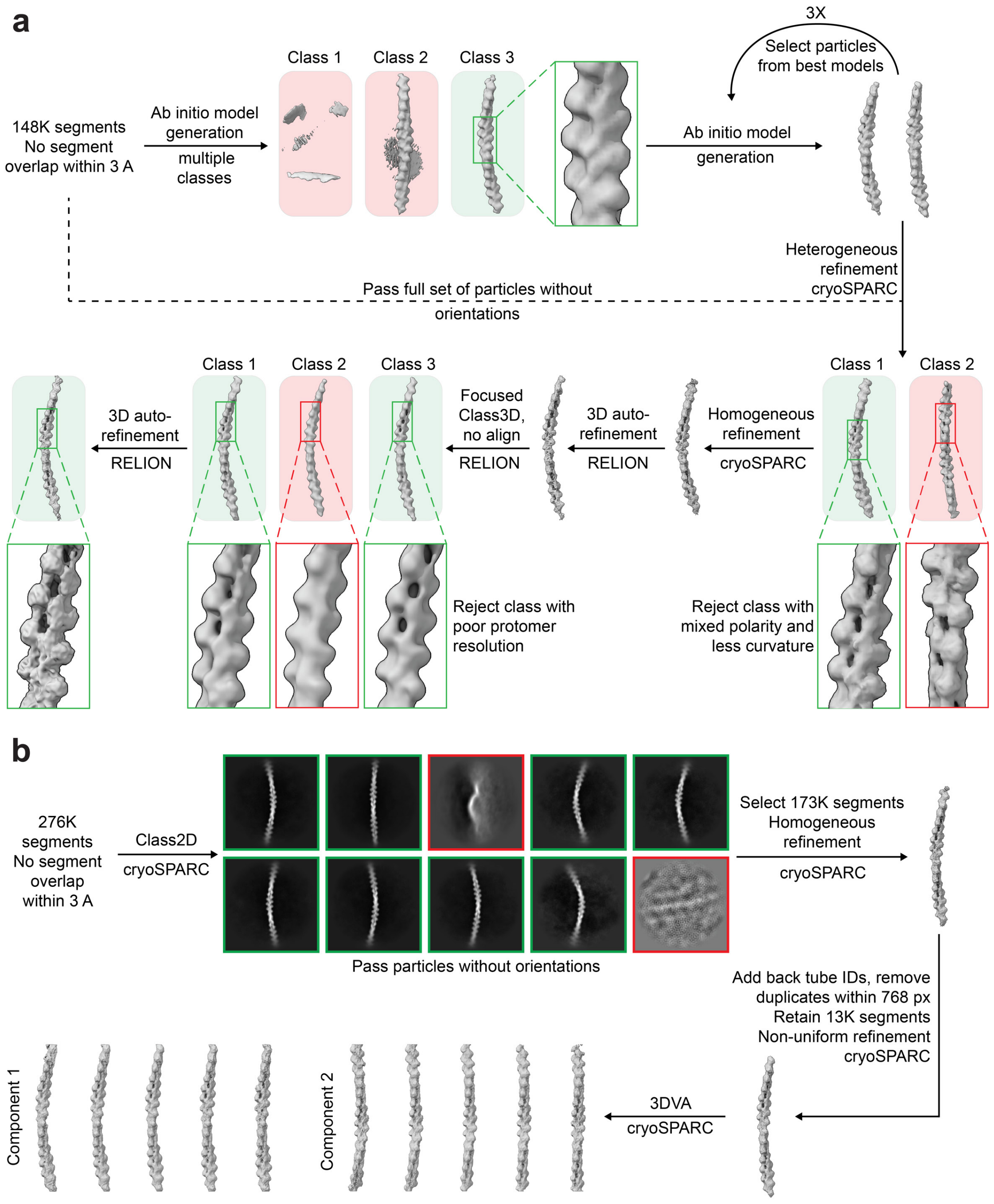

Fig. S4: Superhelix structure determination workflow and variability analysis.
a, Initial single particle cryo-EM data processing workflow for superhelical F-actin, using a single dataset collected for the dual motor condition. Transparent red and green boxes indicate rejected and accepted classes, respectively. **b**, Final processing workflow, incorporating data from two additional datasets to boost particle number and enhance the quality of the final map. Additionally, 3DVA variability analysis is displayed, highlighting the presence of continuous structural variability despite extensive classification.

bioRxiv preprint doi: https://doi.org/10.1101/2024.08.15.608188; this version posted August 17, 2024. The copyright holder for this preprint (which was not certified by peer review) is the author/funder, who has granted bioRxiv a license to display the preprint in perpetuity. It is made available under aCC-BY 4.0 International license.

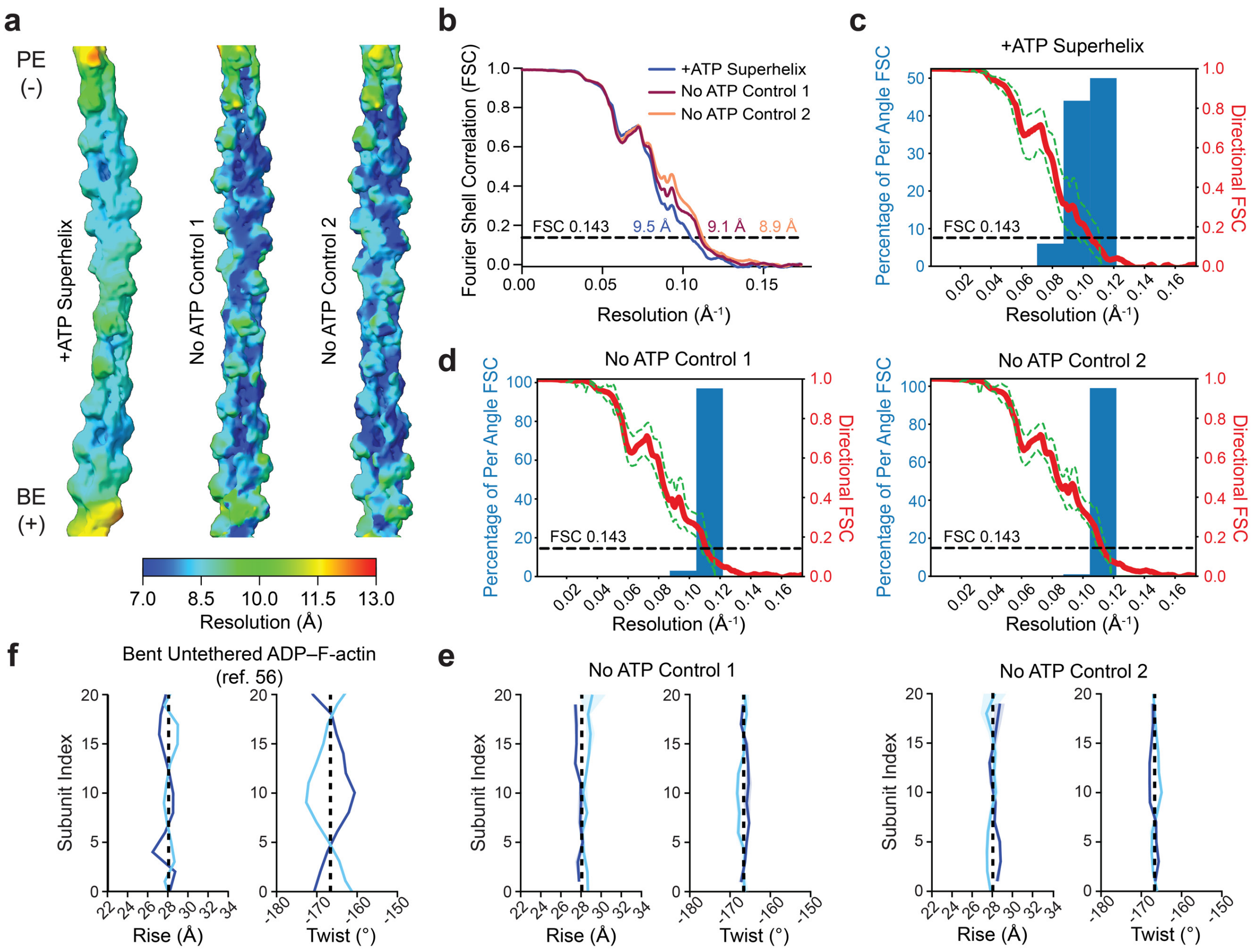

Fig. S5: Resolution assessment and helical parameter analysis of superhelical and control F-actin. **a**, Local resolution of +ATP myosin-force evoked superhelical F-actin (left) and two independently reconstructed –ATP control maps (right). BE: barbed end; PE: pointed end. **b**, Global Fourier Shell Correlation (FSC) curves. **c and d**, 3DFSC curves for +ATP superhelical F-actin (**c**) and –ATP control reconstructions (**d**). **e**, Instantaneous helical parameters of –ATP control reconstructions, colored as in Fig. 3b. Shaded regions represent 95% CI from 3 independent analyses. **f**, Instantaneous helical parameters of bent, untethered ADP–F-actin with 5.4 μm^{-1} curvature, from ref. 56. Vertical dashed lines indicate parameters of canonical F-actin (ref. 56).

bioRxiv preprint doi: https://doi.org/10.1101/2024.08.15.608188; this version posted August 17, 2024. The copyright holder for this preprint
(which was not certified by peer review) is the author/funder, who has granted bioRxiv a license to display the preprint in perpetuity. It is made
available under aCC-BY 4.0 International license.

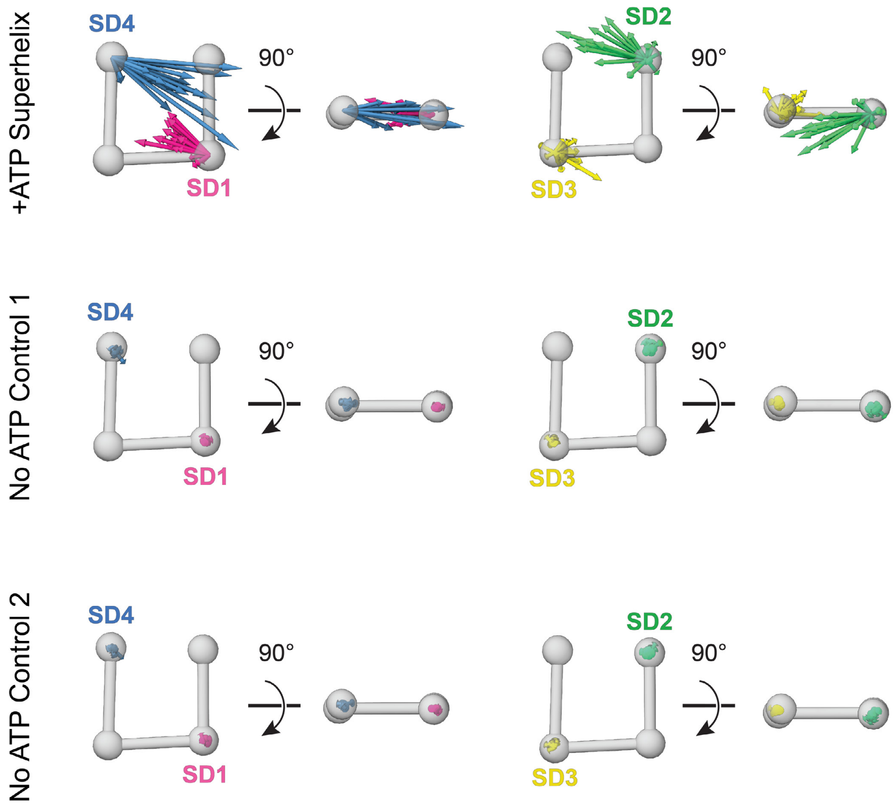

Fig. S6: Subdomain displacements in superhelical F-actin.
Superimposed subdomain displacement vectors from all protomers after MDFF analysis of +ATP myosin force-evoked superhelical F-actin (top) and –ATP control reconstructions (bottom). Subdomains 1 and 4 versus 2 and 3 are displayed separately for clarity, and vectors are scaled 15X for visualization. The averages of these vectors are displayed in Fig. 3c.

bioRxiv preprint doi: https://doi.org/10.1101/2024.08.15.608188; this version posted August 17, 2024. The copyright holder for this preprint (which was not certified by peer review) is the author/funder, who has granted bioRxiv a license to display the preprint in perpetuity. It is made available under aCC-BY 4.0 International license.

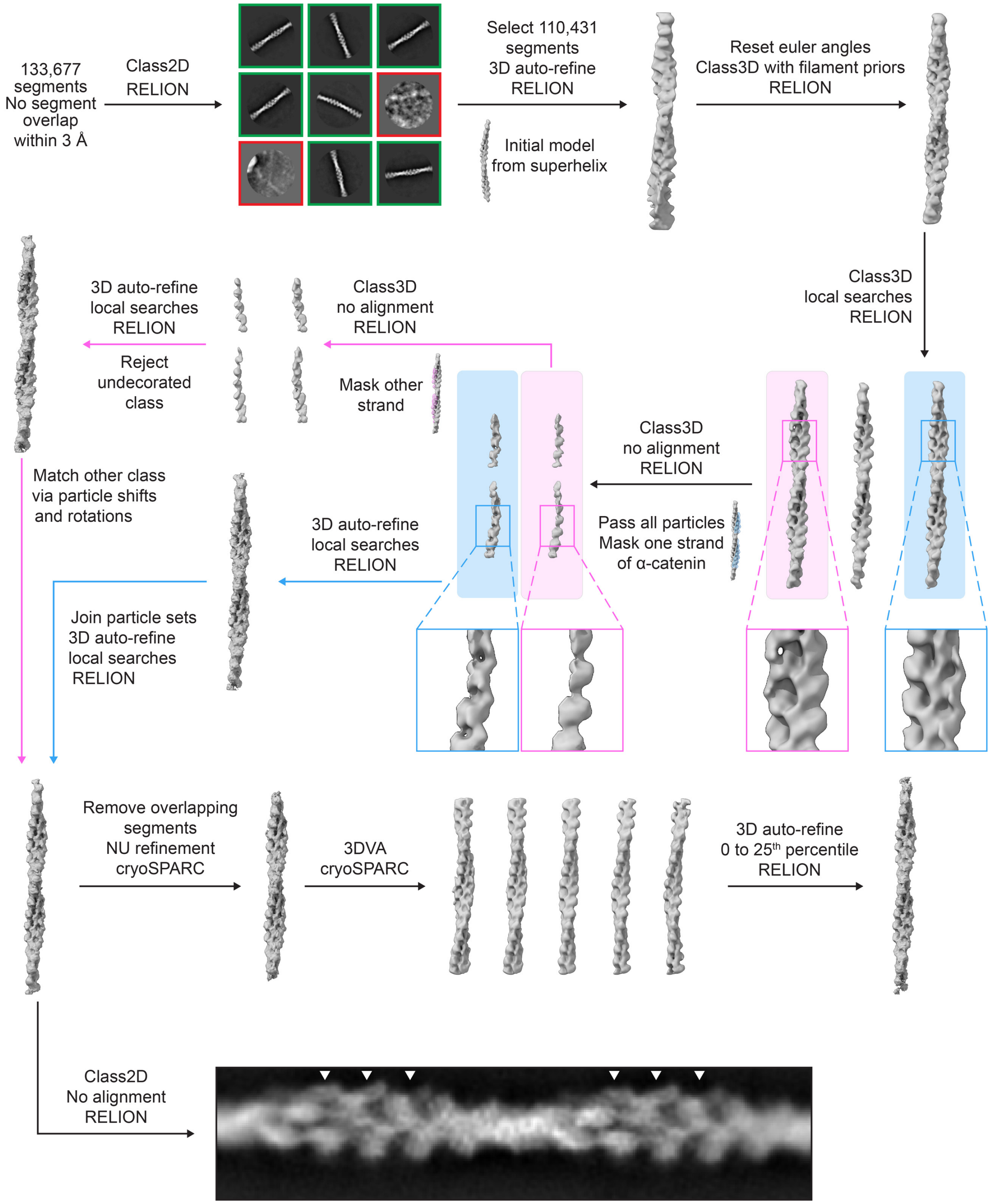

Fig. S7: Cryo-EM processing workflow for the myosin force-activated α-catenin–F-actin complex. Top: Cryo-EM processing workflow for visualizing the force-activated α-catenin-F-actin complex, from a specimen prepared in the dual motor condition. Green and red boxes represent 2D class averages which were selected and rejected for additional processing, respectively. Bottom: a magnified 2D class average is displayed, highlighting preferential binding of α-catenin along one side of the filament (arrowheads) on alternating F-actin strands.

bioRxiv preprint doi: https://doi.org/10.1101/2024.08.15.608188; this version posted August 17, 2024. The copyright holder for this preprint (which was not certified by peer review) is the author/funder, who has granted bioRxiv a license to display the preprint in perpetuity. It is made available under aCC-BY 4.0 International license.

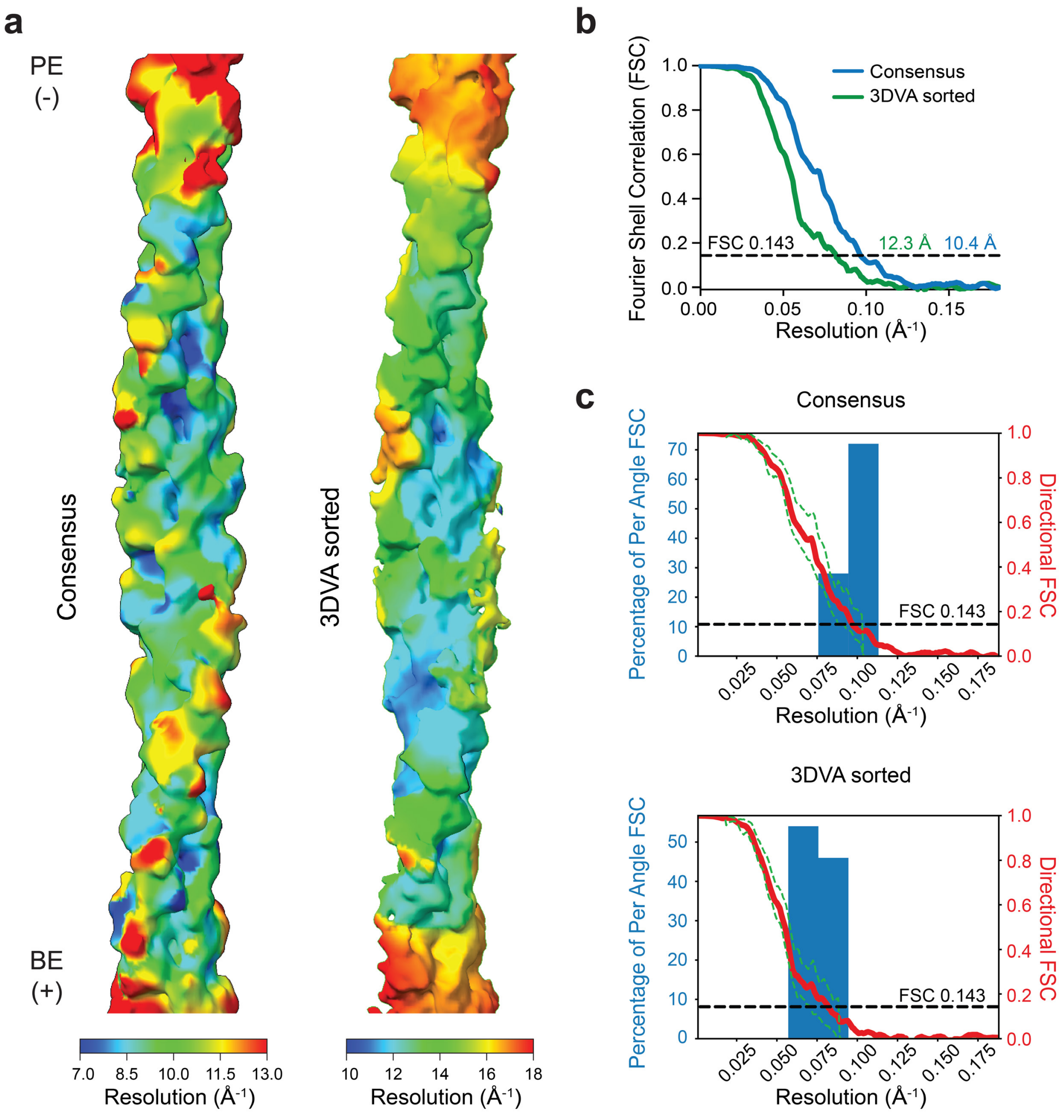

Fig. S8: Resolution assessment of force-activated α-catenin–F-actin complex reconstruction. **a**, Local resolution of final consensus (left) and 3DVA sorted (right) α-catenin–F-actin complex reconstructions. BE: barbed end; PE: pointed end. **b**, Global FSC curves. **c**, 3DFSC curves.

bioRxiv preprint doi: https://doi.org/10.1101/2024.08.15.608188; this version posted August 17, 2024. The copyright holder for this preprint (which was not certified by peer review) is the author/funder, who has granted bioRxiv a license to display the preprint in perpetuity. It is made available under aCC-BY 4.0 International license.

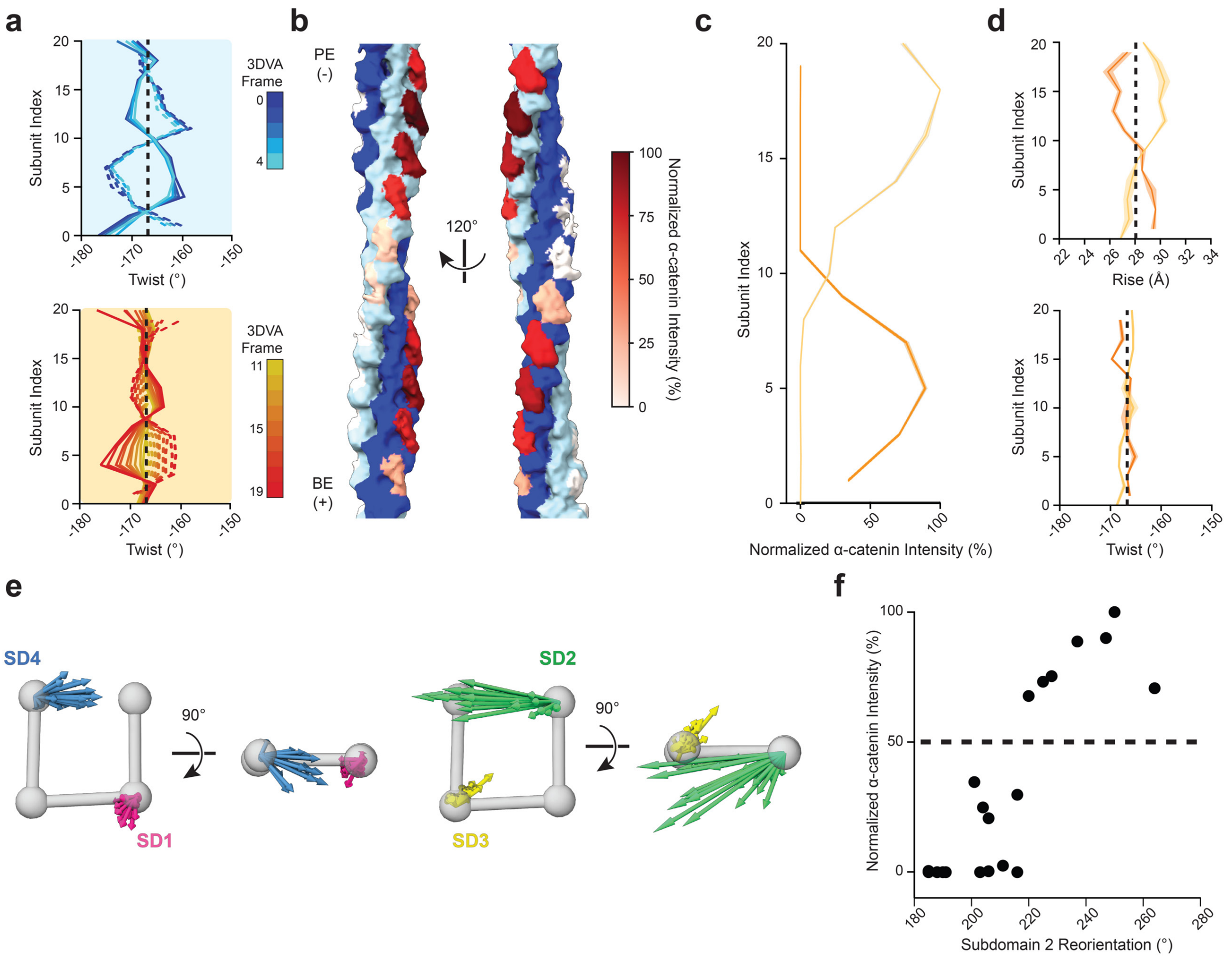

Fig. S9: Additional analysis of the force-activated α-catenin–F-actin complex structure.
a, Instantaneous helical twist of selected 3DVA frames from Fig. 4b,c. Vertical dashed lines indicate canonical F-actin twist. **b**, Orthogonal views of the post-3DVA α-catenin–F-actin complex map, colored as in Fig. 4a. **c**, Quantification of α-catenin density intensity in post-3DVA map. Yellow corresponds to light blue strand from a, and orange corresponds to dark blue strand. **d**, Instantaneous helical parameters of consensus map, colored as in b. Shaded regions represent 95% CI from 3 independent analyses. Vertical dashed lines indicate parameters of canonical F-actin. **e**, Superimposed subdomain displacement vectors versus a canonical F-actin subunit from all protomers after MDFF analysis of the consensus α-catenin–F-actin complex map, displayed as in Fig. S6. The averages of these vectors are displayed in Fig. 4f, top. **f**, Quantification of actin subdomain 2 reorientation versus α-catenin intensity. Dashed line indicates 50%.

bioRxiv preprint doi: https://doi.org/10.1101/2024.08.15.608188; this version posted August 17, 2024. The copyright holder for this preprint (which was not certified by peer review) is the author/funder, who has granted bioRxiv a license to display the preprint in perpetuity. It is made available under aCC-BY 4.0 International license.

Table S1. Cryo-EM data collection and processing statistics

**Data collection**							
Microscope	Titan Krios						
Voltage (kV)	300						
Sample	Dual Motor +ATP			Dual Motor -ATP		Dual Motor +ATP + α-catenin	
Detector	K3	K3	K3	K3		K2 summit	
Magnification	64,000	64,000	64,000	64,000		22,500	
Electron exposure ($e^-/Å^2$)	59.156	59.156	59.156	59.156		56.53	
Exposure rate (e^-/pixel/s)	23.66	23.66	23.66	23.66		5.65	
Calibrated pixel size (Å)	1.08	1.08	1.08	1.08		1.33	
Defocus range (μm)	-1.5 to -3.5	-1.5 to -3.5	-1.5 to -3.5	-1.5 to -3.5		-1.5 to -3.5	
Micrographs (No.)	12,453	10,842	8,504	3,749		4,455	
**Data Processing**	Dual Motor +ATP			-ATP Control 1	-ATP Control 2	Consensus	3DVA sorted
Initial particles (No.)	276,493			345,515		133,677	
Final particles (No.)	13,146			3436	3137	3,289	822
Symmetry imposed	C1			C1	C1	C1	C1
Map resolution (Å)	9.5			9.1	8.9	10.4	12.3
FSC threshold	0.143			0.143	0.143	0.143	0.143
Map sharpening B factor ($Å^2$)	-886.6			-850.8	-801.5	-1151.9	-100.0

Table S2. Coarse grained molecular dynamics simulation parameters

$k_{l\ axial}$ (pN/nm)	265
$k_{l\ lateral}$ (pN/nm)	53
$k_{\theta 1}$ (kJ)	1.2×10^{-15}
$k_{\theta 2}$ (kJ)	2.4×10^{-15}
$k_{\theta 3}$ (kJ)	1.2×10^{-15}
$k_{\theta 4}$ (kJ)	6×10^{-16}
$k_{\theta 5}$ (kJ)	6×10^{-16}
$k_{\theta 6}$ (kJ)	3×10^{-16}
$k_{\phi 1}$ (kJ)	1.5×10^{-16}
$k_{\phi 2}$ (kJ)	7.5×10^{-16}

bioRxiv preprint doi: https://doi.org/10.1101/2024.08.15.608188; this version posted August 17, 2024. The copyright holder for this preprint (which was not certified by peer review) is the author/funder, who has granted bioRxiv a license to display the preprint in perpetuity. It is made available under aCC-BY 4.0 International license.

Supplementary Video Legends

Video S1: Cryo-ET of a Ptk2 cell adhesion marked by zyxin. Segmentation is colored as in Fig. 1b.

Video S2: Reconstituting myosin motility on cryo-EM grids. Unanchored actin filaments were visualized by rhodamine-actin fluorescence (blue) in the presence of the indicated motors, while the holey carbon substrate was visualized by brightfield (grey).

Video S3: Dynamics of dual motor assay on cryo-EM grids. Actin filaments were visualized by rhodamine-actin fluorescence (blue) in the absence and presence of ATP to activate motors. Arrowhead indicates a mechanically-induced severing event. The substrate was visualized by brightfield (grey).

Video S4: Dynamics of single motor assays on cryo-EM grids. Actin filaments anchored through biotinylated seeds were visualized by rhodamine-actin fluorescence (blue) in the presence of either myosin-6 (“pointed end”) or myosin-5 (“barbed end”). Arrowheads indicate mechanically-induced severing events. The distribution of myosins was visualized by eGFP fluorescence (grey), which marks the substrate.

Video S5: Cryo-ET of superhelical oscillatory domain. Serial slices through a tomogram from the barbed end directed force condition. Scale bar, 50 nm.

Video S6: Coarse-grained MD simulations mimicking motor dynamics elicit F-actin spirals. Movie of the time evolution of simulations presented in Fig. 2f.

Video S7: Reconstruction of superhelical F-actin. Movie of stitched superhelix reconstruction (colored as in Fig. 3a), highlighting the three-dimensional corkscrewing character of the filament.

Video S8: 3DVA captures coupling between F-actin remodeling and α-catenin engagement. Movie of 3DVA frames, highlighting both changes in filament curvature and α-catenin binding. α-Catenin is colored by normalized intensity (a proxy for occupancy) as in Fig. 4a.
